# Supplementary figures and images for: Sensory stimuli dominate over rhythmic electrical stimulation in modulating behavior
Source: PLoS Biol. 2025 Jun 5;23(6):e3003180. doi: 10.1371/journal.pbio.3003180 (PMC12140215; doi:10.1371/journal.pbio.3003180)

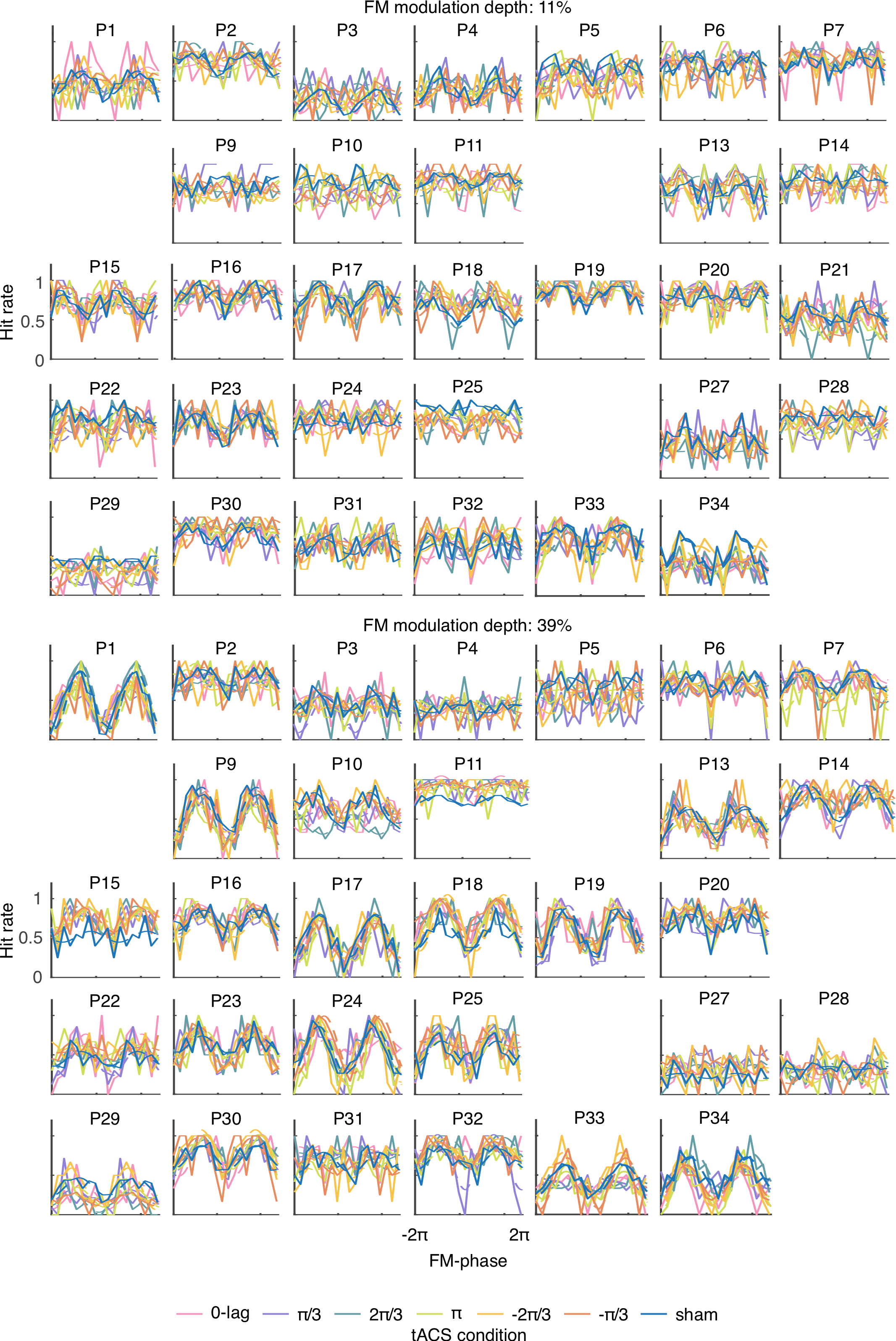

Supplement: S1 Fig — Supplementary figure accompanying Fig 1D. Individual data showing hit rates as a function of FM-stimulus phase for each tACS condition when the FM stimulus is modulated at 11% (top) and 39% (bottom). Each plot shows data from a different participant and modulation depth. Empty plots represent participants excluded due to incomplete data. (TIF) [file pbio.3003180.s001.tif]

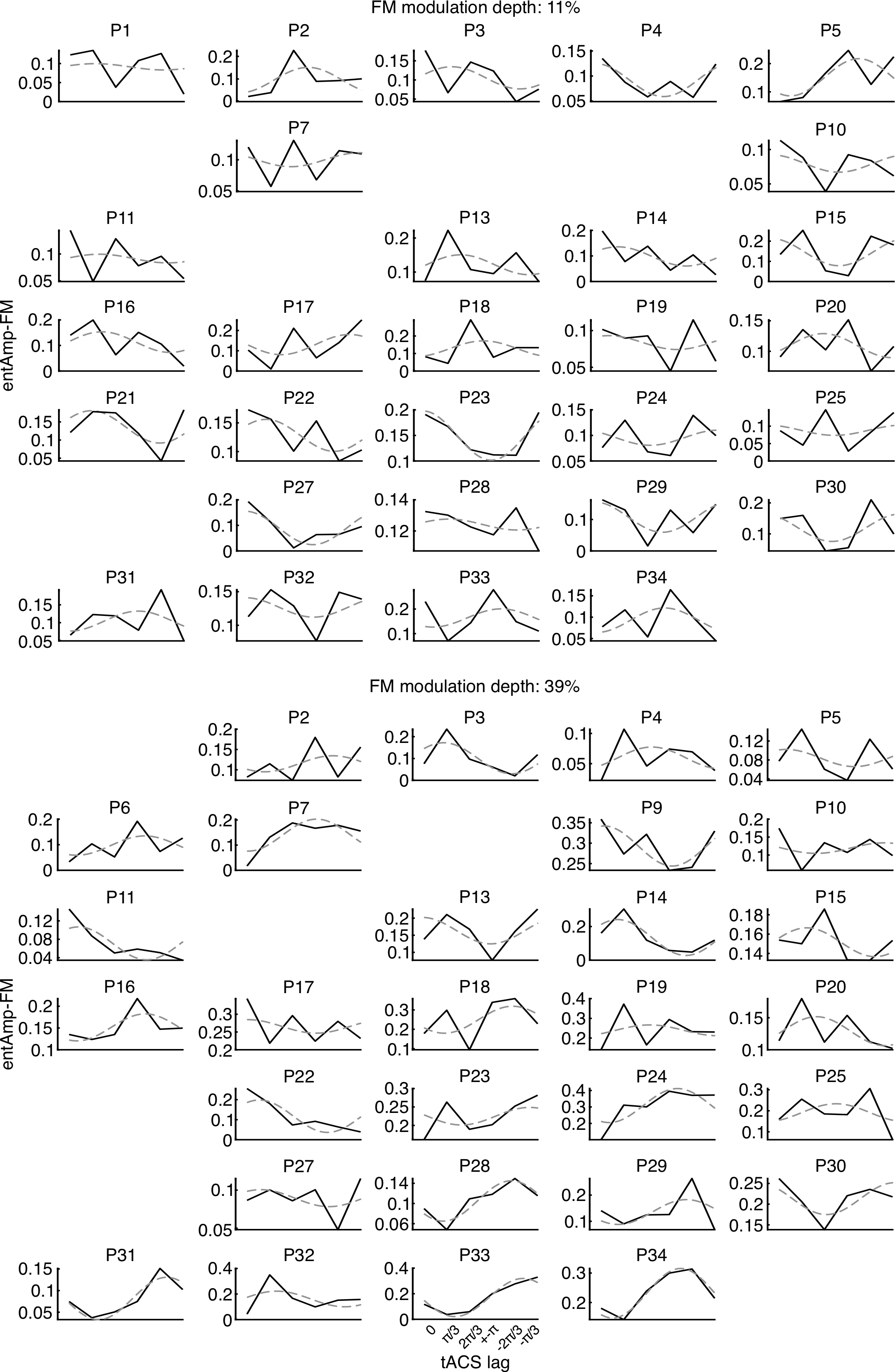

Supplement: S2 Fig — Supplementary figure accompanying Fig 1I. EntAmp-FM as a function of tACS lag for each modulation depth; 11% (top) and 39% (bottom). Solid lines show the actual amplitude parameters obtained from the initial cosine fits on the data in Fig 1D and dashed lines represent the second cosine fit to estimate the optimal tACS phase for modulating entrainment to the auditory stimulus. Each plot is a different participant and modulation depth. Empty plots represent participants excluded due to incomplete data. (TIF) [file pbio.3003180.s002.tif]

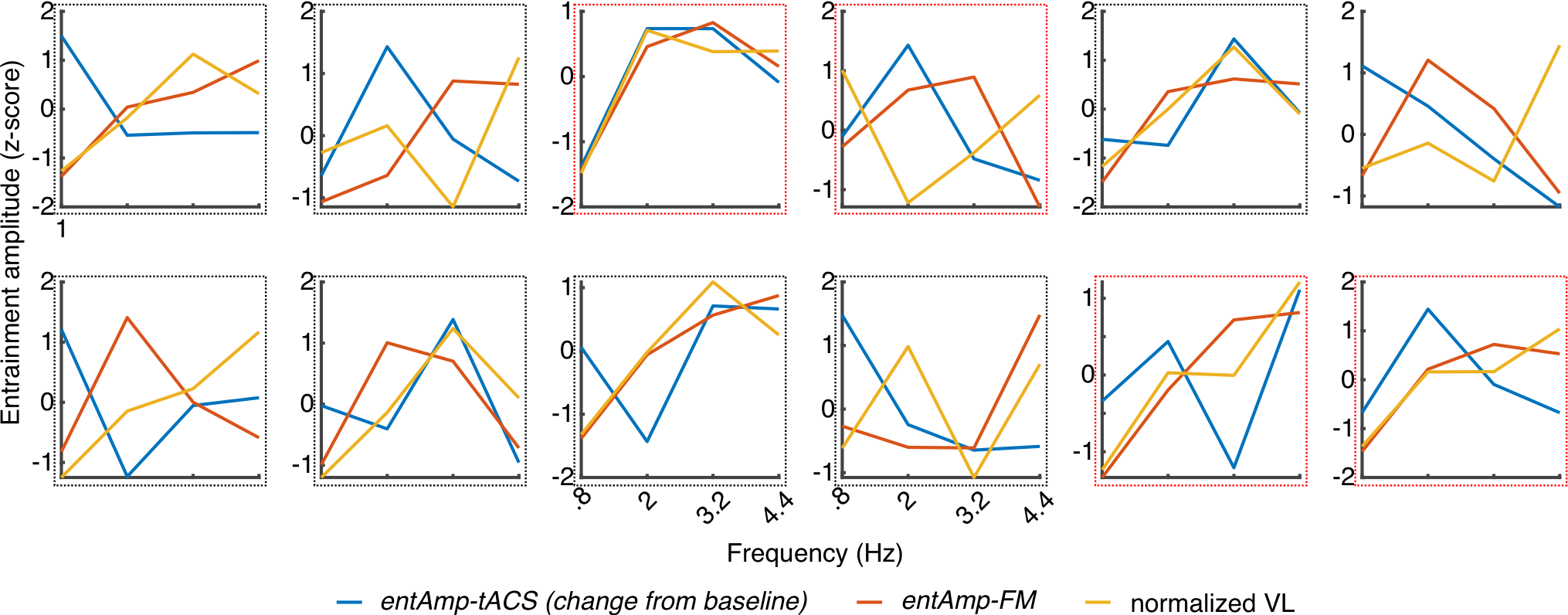

Supplement: S3 Fig — The plots display entrainment amplitude as a function of frequency for Experiments 2 and 3. In Experiment 2, behavioral entrainment to tACS is quantified as (entAmp-tACS − baseline)/baseline, while in Experiment 3, behavioral and neural entrainment to FM sounds are represented by entAmp-FM and normalized vector length (VL), respectively. Data is shown for the 12 participants who took part in both experiments. Black dashed squares highlight participants with a tACS effect greater than 1 for at least one frequency. Red dashed squares indicate participants with a positive tACS effect for at least one frequency, but less than 1. (TIF) [file pbio.3003180.s003.tif]

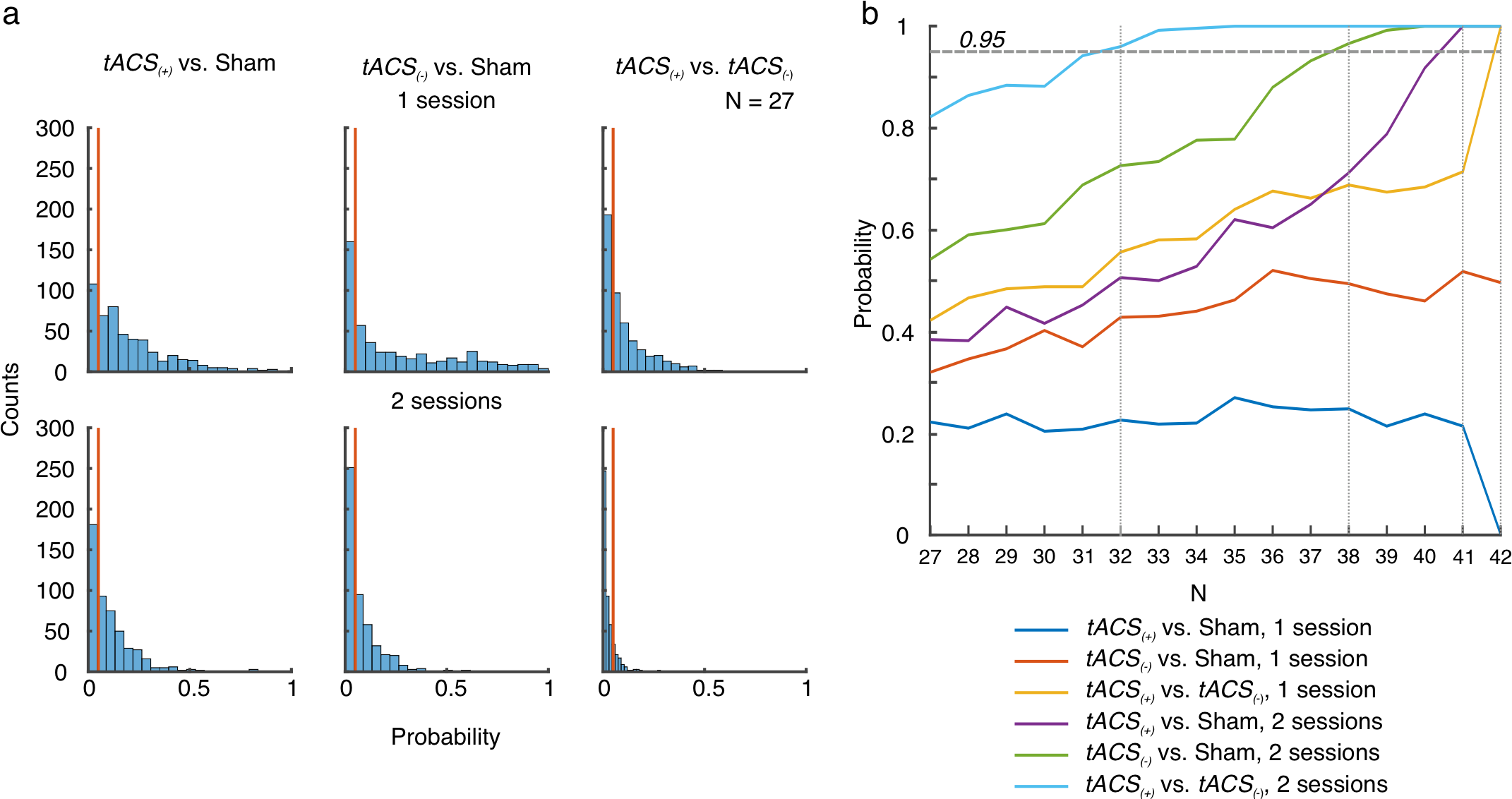

Supplement: S4 Fig — The distributions are shown for sample of N = 27 using only one session data or the average across two sessions. (b) Probability of observing a significant tACS effect of each contrast of interest as a function of the sample size (N = 27–42). Note that for N = 42, only one independent sample can be drawn and only 42 different samples could be drawn for N = 41. Similar to (a), the analysis was performed using data from only one session or using the average across two sessions. (TIF) [file pbio.3003180.s004.tif]
