# Supplementary material for: Sensory stimuli dominate over rhythmic electrical stimulation in modulating behavior
Source: PLoS Biol. 2025 Jun 5;23(6):e3003180. doi: 10.1371/journal.pbio.3003180 (PMC12140215; doi:10.1371/journal.pbio.3003180)
Supplement: S1 Table — Δ AIC relative to winning model. The winning model is also highlighted in bold. AIC, Akaike information criterion. (DOCX) [file pbio.3003180.s005.docx]

**Table S1. Mixed effects logistic regression models explaining single trial gap detection performance in Experiment 1**

|  | Formula | AIC | Δ AIC* |
| --- | --- | --- | --- |
| **1** | ***response ~ 1 + sinAUD + cosAUD + (1 \| subjectNr)*** | 33917.95 | 0 |
| 2 | *response ~ 1 + sintACS + costACS + sinAUD + cosAUD + (1 \| subjectNr) + (1 + sintACS \| subjectNr) + (1 + costACS \| subjectNr)* | 33922.61 | 4.66 |
| 3 | *response ~ 1 + sinAUD*modDepth + cosAUD*modDepth + (1 \| subjectNr)* | 33923.95 | 6.00 |
| 4 | *response ~ 1 + sinAUD + cosAUD + sintACS*modDepth + costACS*modDepth + (1 \| subjectNr) + (1 + sintACS \| subjectNr) + (1*  *+ costACS \| subjectNr)* | 33928.61 | 10.66 |
| 5 | *response ~ 1 + sintACS + costACS + (1 \| subjectNr) + (1 + sintACS \| subjectNr) + (1 + costACS \| subjectNr)* | 34787.58 | 869.63 |

Models are organized from smallest to highest AIC. Δ AIC relative to winning model. The winning model is also highlighted in bold. AIC, Akaike information criterion
