# Supplementary material for: Sensory stimuli dominate over rhythmic electrical stimulation in modulating behavior
Source: PLoS Biol. 2025 Jun 5;23(6):e3003180. doi: 10.1371/journal.pbio.3003180 (PMC12140215; doi:10.1371/journal.pbio.3003180)
Supplement: Attachment — Submitted filename: Response_letter.docx [file pbio.3003180.s008.docx]

**Yuranny Cabral-Calderin**
Max Planck Institute for Empirical Aesthetics
60322, Frankfurt am Main, Germany
E-mail: yuranny.cabral-calderin@ae.mpg.de
Tel: 49 69 8300479-831
December 30^th^, 2024

**Editorial Board**

Plos Biology

Dear Dr. Schnell, dear Editors,

Thank you for organizing the reviews for our manuscript, *"Superiority of Rhythmic Auditory Signals over Electrical Stimulation to Entrain Behavior,"* and for the invitation to submit a revision. We would also like to thank the reviewers for their constructive feedback and collegial tone. Addressing their comments was a rewarding process, as their insights and guidance enhanced the quality of the manuscript. We also appreciate the reviewers’ balanced evaluation, which not only highlighted areas for improvement but also acknowledged the strengths of our work. This recognition motivates us to continue striving for rigorous and impactful science.

We have addressed every comment, which has helped to improve the transparency and readability of the manuscript. The main changes to the manuscript are summarized as follows:

1. **Expansion of Experimental Design Details**

- We have expanded the information about the experimental design to clarify that our auditory stimuli were **frequency-modulated (FM) sounds**, not **amplitude-modulated (AM)**, emphasizing that amplitude remained constant throughout the study.
- Additional methodological details have been provided to explain how **modulation depth conditions** were randomized across participants.

2. **Figure Modifications Based on Reviewer Feedback**

- **X-axis divisions** were adjusted in key figures (**Figures 1a, 3a, and 5a**) to improve readability.
- **Figure 1** was reorganized, with some panels moved to **a new Figure 2** for better clarity.
- A new panel was added to **Figure 1** to display **false alarm rates**, and figure legends were updated for accurate interpretation.
- **New panels** were added to **Figure 2 and Figure 4** to better illustrate **methodological details** related to the **realignment procedures** and **computational model**.

3. **Additional Analyses Conducted as Requested**

- **Bayes factors** are now reported for **non-significant findings** to distinguish between inconclusive results and evidence supporting the null hypothesis.
- **False alarm rates** were analyzed and incorporated into the results.
- The explanation of **realignment procedures** and **statistical methods** used for data analysis has been improved.
- The probability of a significant tACS effect as a function of sample size has been estimated using the preprocessed data from our previous study (Cabral-Calderin et al., 2024).

4. **Expanded Discussion**

- We have strengthened key discussion points, including:
  - The **comparison between auditory and electrical stimulation entrainment**.
  - **Individual variability** in optimal entrainment frequency and its potential implications for future research.

5. **Overall Manuscript Revisions**

- The manuscript has been revised to improve **clarity, readability, and consistency** based on reviewer feedback.

Below, you will find a point-by-point response to the reviewers’ comments. Our responses are in **BOLD.** We have reproduced new and edited sections of the text here for your and the reviewers’ convenience (in *italics*). The new and edited text in the manuscript file is highlighted using the “track-changes” option of Microsoft word. Note that the exact pages and lines referenced below may vary slightly from those in the track-changes document due to different features of Microsoft Word.

Thank you again for your consideration.

Sincerely,

Yuranny Cabral-Calderin & Molly J Henry

Response to Reviewers’ comments

Reviewer #1 (Heather L Read): Title: "Superiority of Rhythmic Auditory Signals over Electrical Stimulation to Entrain Behavior"

EXCELLENT RATIONALE. Prior studies have shown that transcranial stimulation can modulate sensory stimulus-brain synchrony and auditory detection performance. Accordingly, the authors previously found that tACS could enhance or diminish entrainment to auditory stimulation, depending on the phase relationship between auditory and electrical signals ( Cabral-Calderin et al., 2023). Yet the auditory stimuli played a dominant role in guiding behavior on a trial-by-trial basis. This leads the team to ask how auditory and electrical entrainment of brain rhythms may interact or compete.

HIGH SIGNIFICANCE & NOVELTY. This elegant study demonstrates that rhythmic frequency and amplitude modulated sounds are more effective at entraining slow delta oscillations than transcranial electrical stimulation (aka, tACS). Moreover, this study demonstrates that slow delta oscillations are more likely to be entrained to tACS when the frequency for stimulation is tailored to the individual's optimal entrainment frequency. A similar principle applies to the entrainment of intrinsic oscillations with natural rhythmic sensory stimulation. Thus, this study suggests a novel general principle where effective entrainment requires electric or sensory stimulus frequencies to be tailored (optimized) to the individual's intrinsic oscillatory frequencies. Tailoring entrainment with natural sensory stimuli or electrical stimuli like tACS has the potential to improve current therapeutic applications.

APPROPRIATE computational Models & Data Analyses.

PUBLICATION SUITABLE with minor edits.

DATASHARING via github page is provided with MATLAB code.

MANUSCRIPT is organized and written well.

METHODS details are sufficient for this study to be reproduced.

DETAILED SUMMARY:

Experiment-1. Participants listened to frequency (FM) and amplitude (AM) modulated sounds that were modulated at 2 Hz with either a small (11%) or a large (39%) amplitude modulation depth, while tACS was applied. Based on their prior study, they hypothesized that the tACS effect should be strongest for the small (11%) FM/amplitude modulated auditory stimuli.

**RE: We would like to point out that all experiments including rhythmic auditory stimulation (i.e., experiments 1 and 3) included only frequency modulation (FM) sounds. Modulation depth is always with respect to the FM stimulus and refers to the distance (in %) between the peak and trough carrier frequencies relative to the center carrier frequency of the sound. Amplitude values were kept constant for all sounds, which was individualized per subject based on their perceptual hearing threshold. We have made the point that the amplitude was constant more explicit in the methods section:**

*Pages 30-31, lines 1033-1035: “Note that the sounds were modulated exclusively in the frequency domain, with no rhythmic information conveyed through the amplitude parameter, which remained constant throughout each session”.*

Result. Behavioral entrainment to the sound (entAmp-FM) was stronger for the large (39%) versus small (11%) modulation depth of sound (t (26) = 126 2.39, p = 0.024, Cohen's d = 0.63, Fig. 1e). Thus, behavioral modulation induced by rhythmic auditory stimulation varies with the sound's amplitude modulation depth.

tACS effects did not differ across amplitude modulation depths as hypothesized.
As in their prior study, they observed a significant boost in behavioral entrainment when tACS was optimally aligned with the stimulus rhythm. However, no significant interaction was observed across electrical and sound stimuli (F (2, 52) = 0.10), p = 0.901), suggesting that the effect of tACS was not significantly different across the amplitude modulation depths.

Experiment-2. Applied tACS at different frequencies while participants listened to unmodulated noise sounds and asked whether tACS could modulate perception when no rhythmic information was conveyed by the sound. They found that individuals varied in the optimal entrainment frequency to tACS in the absence of rhythmic sound variation, this revealed individual variability in preferred frequency for entrainment to electrical stimulation (i.e tACS). Their computational "oscillator" model further supports this idea. Prior work has shown that intrinsic synchronous oscillations are more likely to entrain to external rhythmic sensory input when the rhythm matches the individual's intrinsic neural oscillator frequency (e.g Notbohm et al., 2016). Thus, this study supports a general principle where entrainment of intrinsic neural oscillators is optimal when the frequencies are aligned to the individual's intrinsic oscillation frequency. They propose that future studies should deliver tACS frequency stimulation tailored to the individual's resonant oscillatory frequencies to potentially improve entrainment, therapeutic settings, and effects.

Experiment-3. Collected EEG data while participants listened to FM sounds with different modulation frequencies, exploring individual variability in neural and behavioral entrainment to acoustic signals in the absence of electrical stimulation.

Overall, acoustical rhythms were more potent in driving behavioral modulation with tACS serving as a supplementary modulator contingent on individual frequency preferences.

MINOR EDIT SUGGESTIONS

1. Fig. 1a. The x-axis has 5 divisions over 6 seconds of sound. Would be easier to quickly understand the modulation frequency if you had 6 divisions corresponding to 1 sec each.

**RE: Thank you to the reviewer for pointing this out. We agree with the suggestion and have modified Figure 1a accordingly. We have now added six divisions, each corresponding to 1 second. The same adjustments were made to Figures 2a and 4a, which illustrate the stimulus information for experiments 2 and 3, respectively.**

1. Methods. You explain that 3-to-5 silent gaps with a mean duration of 14 (+3.7) ms were separated by > 1.5 seconds within each 20-second long sound segment. Could you please report the mean and standard deviation of the gaps rate (i.e. gaps/sec)?

**RE: we have computed the gaps rate (gaps/sec) for each experiment and reported it accordingly in the Methods section**

*Experiment 1*

*Page 30, lines 1029-1030: “(average gap rate 0.21 gaps/s, sd = 0.04)”*

2. Lines 129-132. "During the task, hit rates were significantly higher for the 11% condition than for the 39% condition (t (26) = 3.72, p = 9.653e-04, Cohen's d = 0.75, Fig. 1g), suggesting that the task was easier for the 11% condition despite having similar gap sizes." Presumably, hit rates is the detection of gaps throughout the sound segment. In theory, the probability that the gap falls during a loud amplitude modulation is higher with the 39% amplitude modulation condition. So this is surprising. Would be good to explain a possible reason for this result. Is it attentional "competition" between the FM/AM modulation cues and the silent gap detection?

**RE: We believe there is a misunderstanding regarding the characteristics of the stimuli. All stimuli are frequency-modulated (FM) with constant amplitude throughout each 20-second segment and within a given session. The 11% and 39% modulation depths refer to the strength of the FM modulation, specifically the percentage distance between the carrier frequency at the peak/trough of the FM and the stimulus center frequency. We clarified this in our response to the summary of Experiment 1 above and made it more explicit in the Methods section of the paper. The higher hit rates observed in the 11% modulation depth condition compared to the 39% condition could simply be due to the fact that the 11% sound exhibits smaller fluctuations, making it easier to detect the silences. We have added a sentence about it in the results section:**

*Page 6, lines: 134-139: “By definition, the 11% sound exhibits smaller fluctuations compared to the 39% sound; that is, the peak carrier frequency is closer to the stimulus center frequency. Therefore, the higher hit rate observed in the 11% condition suggests that it is easier to detect an auditory event in a more stable environment, where the background sound undergoes less salient frequency changes.”*

3. Lines 370-372. "...averaged cluster of fronto-central electrodes (F3, Fz, F4, FC1, FCz, FC2, C3, Cz, and C4), in line with our previous study characterizing neural entrainment in the auditory system" Please cite your paper Cabral-Calderin & Henry J Neuro 2022 or the correct paper here.

**RE: thanks for the remark. We have cited our paper accordingly (Cabral-Calderin & Henry J Neuro 2022).**

4. DISCUSSION, Lines 497-499. "...the mean strength of behavioral entrainment observed in Experiment 1 with modulation depths of 11% and 39% was smaller than that observed in Experiment 3 and our previous work (modulation depth of 67%, compare Figs. 1e and 5d)." Please include the citation here, given your group has multiple studies on this topic.

**We have cited our paper accordingly (Cabral-Calderin et al., eLife 2024).**

5. DISCUSSION, Lines 501-504. "Previous research has shown that more strongly modulated FM signals elicit stronger neural responses [35]. This effect may occur because stronger FM signals lead to wider coverage of the frequency-tuned neuronal populations activated by the sound, due to the tonotopic organization of the auditory system. This effect may occur because stronger FM signals lead to wider coverage of the frequency-tuned neuronal populations activated by the sound, due to the tonotopic organization of the auditory system. Suggested EDIT. You could expand or revise this this to explain that columns of auditory cortical neurons that are "broadly tuned to sound frequency respond to a broader range of tonal frequencies with higher sound levels" (citations: Read HL, Winer JA, Schreiner CE. Modular organization of intrinsic connections associated with spectral tuning in cat auditory cortex. PNAS, 2001) and these same neurons respond with faster onset-offset responses to dynamic frequency and amplitude modulated sounds (Atencio CA, Schreiner CE. Spectrotemporal processing in spectral tuning modules of cat primary auditory cortex. PLoS One. 2012). Hence, they may track amplitude modulations more accurately. In theory, the FM sounds with larger amplitude modulation depths used here could more strongly activate the broadly tuned columns that follow temporal modulations well.

**RE: We would like to thank the reviewer for the suggestion and for providing the relevant literature. We refrain from incorporating the amplitude modulation discussion, as our stimuli were modulated only in the frequency domain while sound levels were kept constant within each stimulus and session; i.e., no amplitude modulation was used (see response to previous comments). We have incorporated the rest of the suggestion into our discussion in:**

*Pages 21-22, lines 720-726: "Columns of auditory cortical neurons that are broadly tuned to sound frequency respond with faster onset-offset responses to dynamically frequency- and amplitude-modulated sounds (Atencio CA, Schreiner CE. Spectrotemporal processing in spectral tuning modules of cat primary auditory cortex. PLoS One, 2012). Theoretically, the FM sounds with larger modulation depths used here could more strongly activate the broadly tuned columns that efficiently follow temporal modulations."*

6. Lines 629-634. This is an excellent point you make about how we need to explore the potential benefits of individual tailoring of tACS and auditory stimulation to optimize entrainment or other neuromodulations.

**RE: We would like to thank the reviewer for pointing this out. We agree that we need to keep working on individualize stimulation protocols to establish their potential benefits.**


Reviewer #2: In this paper, Cabral-Calderin and Henry, present three experiments investigating entrainment to tACS and auditory rhythms when these are presented simultaneously or in isolation. Across experiments participants were required to detect silent gaps in modulated or unmodulated noise stimuli. They found that when both stimuli were presented simultaneously, auditory stimuli dominated entrainment with no additional effect from tACS. There was evidence of entrainment when tACS was presented in isolation, but the frequency of entrainment varied across participants; similar varied entrainment frequencies were observed when sounds were presented in isolation.

Overall, the quality and rigour of the research presented is high. The manuscript is well written and the experimental protocol and data analysis presented in a clear manner. The paper will be of interest to researchers working on neurostimulation.

I have listed comments and questions below.

**RE: we would like to thank the reviewer for the positive evaluation of our work.**

1. One of the key conclusions of the paper is that when sensory and electrical stimuli compete for entrainment, sensory stimuli is more powerful. This conclusion somehow assumes that we are comparing stimuli of comparable intensities. Indeed, assuming that we are phase resetting an intrinsic oscillator, the amount of phase reset depends on the intensity of the perturbation. For both sound and tACS the intensity was kept constant, 55 dB ad 1 mA, respectively, but it is hard to know if these are 'equivalent'. While the authors do mention this as a limitation it would be relevant to reflect on this more widely, as well as the choice of stimulation intensity given current trends for higher current strengths.

**RE: We appreciate the reviewer’s comment, as it highlights a critical challenge in comparing intensities between the sound and tACS conditions. A major limitation is the absence of a direct measure of tACS efficacy in the auditory system. Unlike the motor or visual systems, which have established proxies such as motor-evoked potentials or phosphene thresholds to gauge cortical excitability, no comparable metrics exist for the auditory system. One potential solution could involve calibrating tACS intensity relative to the individual's detection threshold using skin sensation as an indirect proxy. While skin sensation from tACS does not directly correspond to auditory stimulus thresholds, it could help account for individual variability in perception. Another promising approach, recently proposed for TMS (Numssen et al., 2024), is to define stimulation intensity based on the motor threshold during motor cortex stimulation and estimate the corresponding intensity in the target area through computational modeling. Future research could validate the applicability of this method for tACS. Our decision to employ 1 mA for tACS stimulation was guided by safety considerations, given the electrode size, and tolerability for the average participant. While many tACS studies have employed higher intensities (e.g., 1.5–2 mA), it is important to note that 1 mA has been successfully used in previous studies. These studies demonstrate that tACS can influence auditory perception (Baltus et al., 2018; Cabral-Calderin et al., 2024) and alter brain activity and functional connectivity (Cabral-Calderin, Williams, et al., 2016), as measured with fMRI.**

**We have incorporated these points into the discussion.:**

*Pages 26-27, lines 861-887: “A key challenge in entraining behavior with two different signals is the difficulty of comparing their intensities. Assuming that auditory and electrical signals compete to reset the phase of an intrinsic oscillator, the extent of phase resetting depends on the intensity of the perturbation. While the intensities of both entraining signals were kept constant throughout the experiment, determining whether these intensities are equivalent is challenging. The intensity of the auditory stimulus was individually defined to be 55 dB above the hearing threshold, whereas the intensity of tACS was fixed at 1 mA peak-to-peak for all participants. A major limitation is the lack of a direct measure of tACS efficacy on the excitability of the auditory system. Unlike the motor or visual systems, where proxies such as motor-evoked potentials or phosphene thresholds are used to gauge cortical excitability, no comparable metrics exist for the auditory system. One potential solution could involve calibrating tACS intensity relative to an individual’s detection threshold using skin sensation as an indirect proxy. Although skin sensation from tACS does not directly correspond to auditory stimulus thresholds, it could help account for individual variability in perception. Note that most participants in our study reported to feel the stimulation to some extent, which suggests that the stimulation intensity of 1 mA was above the skin sensation threshold. Another promising approach, recently proposed for TMS (Numssen et al., 2024), involves defining stimulation intensity based on the motor threshold during motor cortex stimulation and estimating the corresponding intensity in the target area using computational modeling. Future research could explore and validate the applicability of this method for tACS. Our choice of 1 mA for tACS stimulation was guided by safety considerations, given the electrode size, as well as tolerability for the average participant. While many tACS studies have utilized higher intensities (e.g., 1.5–2 mA), prior research has demonstrated that 1 mA tACS is also effective for modulating auditory perception (Baltus et al., 2018; Cabral-Calderin et al., 2024) and alter brain activity and functional connectivity (Cabral-Calderin, Williams, et al., 2016), (Cabral-Calderin, Anne Weinrich, et al., 2016). Our findings suggest that auditory rhythms presented well above the hearing threshold are more effective than 1 mA tACS at entraining behavior. Future studies could further investigate this relationship and explore alternative methods to standardize and compare the intensities of auditory and electrical signals.”*

2. Order of modulation depth. The authors state that each modulation depth was tested on a different session with the order randomized across participants. Is this randomized or counter-balanced? Is the order balanced across conditions?

**RE: The order of the modulation depth was randomized across participants. Initially, 18 participants were presented with 11% modulation depth in session one and 16 with 39%. However, only 30 participants performed both sessions; 17 of these were presented with 11% modulation depth stimuli in session 1 and 39% modulation depth in session 2, while the remaining 13 participants experienced the reverse order. Regarding the tACS phase lag condition, this was balance across modulation depths**

*Page 32, lines 1071-1073:* *“(of the 30 participants who took part in both sessions, 17 were presented with 11% modulation depth stimuli in session 1 and 39% modulation depth in session 2, while the remaining 13 participants experienced the reverse order).”*

3. Since entrainment is higher at 39% modulation, shouldn't performance also follow the same pattern? Could you add some explanation for why this is not the case?

**RE: Given the growing body of literature linking entrainment to auditory perception, the reviewer is right in assuming that higher entrainment should be associated with better performance (Brodbeck et al., 2020; Doelling et al., 2014; Doelling & Poeppel, 2015; Golumbic et al., 2013; Henry et al., 2014; Henry & Obleser, 2012; Horton et al., 2014; Nozaradan et al., 2016; Peelle & Davis, 2012). However, this relationship is highly task-dependent. Based on our previous EEG study (Cabral-Calderin & Henry, 2022), we know that neural activity entrains to the FM stimulus. Entrainment of slow oscillations to the FM stimulus rhythm suggests that targets presented during the excitatory phase of the entrained oscillation are better processed than those presented during the inhibitory phase. In our task, gaps are randomly presented at equally spaced bins across the modulation cycle. We characterized behavioral entrainment using the amplitude parameter of a cosine function fitted to the hit rates (performance) as a function of stimulus phase. Higher entrainment in this context reflects a larger difference between hit rates at the peak and trough of the stimulus phase—consistent with neural entrainment to the sound—and is independent of mean performance, which represents hit rates averaged across all phases.**

**Previous literature suggests that gap detection is better in unmodulated than in modulated noise. For example, while gap detection thresholds in unmodulated noise have been reported around 2 and 3 ms for broadband noise in early studies (Eddins et al., 1992; Green & Forrest, 1989; Shailer & Moore, 1985), gap detection thresholds in amplitude modulated sounds seem to be higher, around 8-10 ms (Mizuno et al., 1994) or higher depending on the kind of modulation (Grose et al., 2008). We believe that overall task performance is higher at the 11% modulation depth because the task is easier under this condition. It is easier to detect the silent target within a stimulus that is perceptually more constant (lower modulation depth) than in the strongly modulated 39% condition. As we showed previously (Cabral-Calderin & Henry, 2022), gap detection performance in this paradigm is influenced not only by entrainment but also by alpha power. It is possible that the continuous mode mediated by alpha activity provides a more effective mechanism than neural entrainment for this particular task. We have included this explanation in the discussion section of our paper.**

*Pages 22-23, lines 735-761: “Of note, in our case, higher entrainment to the 39% modulation depth condition did not correspond to higher behavioral performance over all. In fact, average hit rates were higher for the 11% condition than for the 39% condition. Given the growing body of literature linking entrainment to auditory perception, at first glance one would expect that higher entrainment during the 39% modulation depth condition should be associated with better performance (Brodbeck et al., 2020; Doelling et al., 2014; Doelling & Poeppel, 2015; Golumbic et al., 2013; Henry et al., 2014; Henry & Obleser, 2012; Horton et al., 2014; Nozaradan et al., 2016; Peelle & Davis, 2012). However, the relationship between entrainment and auditory perception can be task-dependent. Based on our previous EEG study (Cabral-Calderin & Henry, 2022), we know that neural activity entrains to the FM stimulus. Entrainment of slow oscillations to the FM stimulus rhythm suggests that targets presented during the excitatory phase of the entrained oscillation are better processed than those presented during the inhibitory phase. In our task, gaps are randomly presented at equally spaced bins across the modulation cycle. We characterized behavioral entrainment using the amplitude parameter of a cosine function fitted to the hit rates (performance) as a function of stimulus phase. Higher entrainment in this context reflects a larger difference between hit rates at the peak and trough of the stimulus phase—consistent with neural entrainment to the sound—and is independent of mean performance, which represents hit rates averaged across all phases.*

*Previous literature suggests that gap detection is better in unmodulated than in modulated noise. For example, gap detection thresholds in unmodulated broadband noise have been reported to be around 2–3 ms in early studies [37-39]. In contrast, gap detection thresholds in amplitude-modulated sounds appear to be higher, around 8–10 ms [40], and can vary further depending on the type of modulation [41]. We believe that overall task performance is higher at the 11% modulation depth because the task is easier under this condition. It is easier to detect the silent target within a stimulus that is perceptually more constant (lower modulation depth) than in the strongly modulated 39% condition. As we showed previously (Cabral-Calderin & Henry, 2022), gap detection performance in this paradigm is influenced not only by entrainment but also by alpha power. It is possible that the continuous mode mediated by alpha activity provides a more effective mechanism than neural entrainment for this particular task.*

4. The measure of accuracy used was hit rate, are the levels of false alarms or omissions equivalent between conditions?

**RE: The level of omissions are inversely proportional to the hit rate. Hit rates are computed as the amount of hits/number of targets. Therefore, the significant difference in hit rates also implies a significant difference in the levels of omissions. Based on the reviewers comment, we have now analyzed the levels of false alarms as well. The number of false alarms was not affected by any of the tested conditions in any of the experiments. These results have been included in the manuscript. For experiment 1, we also included a plot in Figure 1, new panel h.**

*Page 6, lines 138-139: “No significant difference was observed for the number of false alarms (t _(26)_ = 0.22, p = 0.829, Bayes factor = 0.208, Fig. 1h).”*

*Page 12, lines 344-345. Repeated measures ANOVAs showed no significant effect of (…) “or the number of false alarms (F (4, 92) = 1.3, p = 0.274)”.*

*Page 19, lines 611-613: “The false alarms rate was low (mean = 6.92 across conditions) and was not affected by the FM rate (linear regression model: R^2^ = 0.0145, Adjusted R^2^ = -0.00696, F-statistic vs. constant model = 0.675, p = 0.416).”*

*Page 41, lines 1330-1332: “Other statistical analyses*

*For each experiment, false alarms were calculated as the number of button presses that were not preceded by a gap.”*

5. Figure 1h - "each row is a different participant". But is it the same participant for each modulation depth? Please clarify in the figure legend. It would perhaps be more relevant to see the same participant for each modulation depth.

**RE: We would like to thank the reviewer for this point. Yes, it is the same participant for each modulation depth. We have clarified this in the figure legend.**

*Page 7, lines 160-161: “Each row represents a different participant, and the same three participants are shown for each modulation depth.”*

6. Figure 2d. I did not understand the continuous signal across frequencies. Please explain this analysis in more detail.

**RE: The continuous signal across frequencies represented the noise floor of behavioral entrainment, i.e., the expected amplitude of periodic fluctuations of gap detection performance independent of tACS. This was estimated by fitting cosine functions to the sham data from each participant, assuming an oscillator resetting at the stimulus onset at a given frequency. This analysis was conducted not only for the frequencies at which tACS was applied but also for all frequencies between 0.1 and 8 Hz, in 0.1 Hz steps, to provide a more comprehensive estimation of the probability of rhythmic fluctuations in behavior across the full delta/theta range. For each frequency, the mean amplitude of imaginary entrainment in the sham condition at that specific frequency, along with the standard error of the mean were shown.**

**Based on the reviewers’ comments, we realized that the figure and analysis description were not clear enough. We have better clarified the analysis and instead of “imaginary entrainment” we now called it “baseline”. We have also removed the plot from the figure and now show instead the change in behavioral entrainment during tACS relative to the baseline, i.e., (entAmp-tACS -baseline)/ baseline, which we hope is more intuitive. See Fig. 3, panel c and e**

*Page 11-12, lines, 328-338: “c) For each tACS frequency, gaps were grouped based on tACS phase. Cosine functions were fitted to the data to estimate the amplitude of behavioral entrainment to tACS. Gap detection performance at sham was used to establish a baseline level of rhythmic fluctuations. We assumed an oscillator that resets at each stimulus onset during sham. Gaps were grouped according to these hypothetical oscillators, and cosine functions were fitted to the resulting data. This was done for the four frequencies used for tACS stimulation. The amplitude parameter from these fits served as the baseline for analyzing tACS effects. In the figure, this is illustrated for the 2 Hz tACS condition. (…) e) Effect of tACS phase on gap detection performance. The plot shows the normalized values computed as (entAmp-tACS-baseline)/baseline, for each frequency.*

7. Figure 3d. Add more information to the legend. What is the x and y axis, what do the colors represent.

**RE: The plot shows a dendrogram from the hierarchical cluster analysis, with the y-axis representing participant numbers and the x-axis indicating the distance between connected pairs. We have updated the figure panel and legend to include this information. Due to several changes in this and other figures, panel 3d is now panel 4e.**

*Page 15, lines 459-460: “Each color represents a different cluster.”*

8. What are the tACS amplitude values for the oscillator model (pg 13, lines 315-316)? Do these represent tACS intensity at different phases?

**RE: tACS amplitude values are independent of phase differences. They represent the stimulation intensity and are relative to the ongoing oscillations amplitude. A value of 0 represents no stimulation at all while a value of 100 represents a tACS signal as strong as the ongoing oscillation. This was done following the same procedure as in (Krause et al., 2022) which states that these values correspond to physiologically relevant conditions. We have made this more explicit in the text:**

*Page 15, lines 469:471: TACS amplitude took on values from 0 to 100% of the baseline oscillation’s amplitude, in steps of 10%, which corresponds to physiologically relevant conditions (Krause et al., 2022).*

9. What instructions were given to participants regarding eyes open / eyes closed?

**RE: Participants were instructed to keep their eyes open throughout the experiment. We have added this information to the methods section.**

*Page 31, lines 1028: “Participants were instructed to keep their eyes open during all tasks.”*

10. Figure 6. The way in which the data is presented in this figure makes it hard to visualize how much individual participants demonstrate similar entrainment frequency preferences across modalities. Perhaps this could be addressed by adding a plot for each of the individuals that took part in experiments 2 and 3 for both modalities showing entrainment per frequency? This could be added to the supplementary.

**RE: We agree with the reviewer that this is a better way to visualize the data. We have removed the previous figure 6 and added the suggested plots to the supplemental figure S3. Moreover, based on this comment and on a comment from Reviewer 3, we have rephrased and condensed the section. The revised section now reads:**

*Page 20, lines 629-641: “Optimal frequency for behavioral and neural entrainment do not overlap*

*We showed that the optimal frequency for entrainment to both tACS and FM stimuli varies across participants. To close the loop, we asked if the preferred frequencies were stable within an individual, regardless of the modality of the rhythmic stimulus. Figure S4 shows the amplitude of behavioral and neural entrainment to tACS and the FM stimulus, respectively, for the 12 participants who took part in both Experiments 2 and 3. While some participants exhibit similar response profiles across tasks and measures, most show different frequency preferences (i.e., the frequency with the highest entrainment) across tasks. This suggests that the optimal entrainment frequency may depend on the stimulation modality. However, note that we tested only four frequencies and included only 12 participants in this analysis. Unfortunately, it was not possible to re-recruit more participants for testing because of the unavailability of participants and relocation of the research group. This remains speculative and requires further investigation with larger sample sizes and measuring more stimulation frequencies.”*

*Supplemental materials, page 6:*

***Fig. S3. Linking individual differences in response profiles across modalities.*** *The plots display entrainment amplitude as a function of frequency for Experiments 2 and 3. In Experiment 2, behavioral entrainment to tACS is quantified as (entAmp-tACS−baseline)/baseline, while in Experiment 3, behavioral and neural entrainment to FM sounds are represented by entAmp-FM and normalized vector length (VL), respectively. Data is shown for the twelve participants who took part in both experiments. Black dashed squares highlight participants with a tACS effect greater than 1 for at least one frequency. Red dashed squares indicate participants with a positive tACS effect for at least one frequency, but less than 1.*

*Pages 28-29, lines 948-951: “When examining individual response profiles across tasks, we observed that while a few participants exhibited similar response patterns across tasks and measures, most displayed different frequency preferences (i.e., the frequency with the highest entrainment) across tasks.”*

Reviewer #3: Yuranny Cabral-Calderin and Molly Henry present a very rich and detailed assessment of entrainment in behavioral and neural (EEG) measures, to auditory stimulation and tACS, in line with their previous work, describing three experiments. Overall, I think this is an important paper, because it sheds light on untested assumptions in the field of neural entrainment, and its careful approach yields some surprising and interesting findings, showing once more that entrainment mechanisms are more complex than initially thought.

The methods are solidly in line with the questions asked, and the results are interpreted very carefully.
Besides the few methodological points that I will list below, my only worry is that the paper is a bit too rich and complex, due to the multitude of findings and the complex methods used, and I therefore encourage the authors to harmonize the methods across the three experiments were possible (see suggestions below).

**RE: we would like to thank the reviewer for the positive evaluation of our manuscript and the thoughtful comments. To track our responses better, we have enumerated the comment below.**

1. Surrogate conditions across experiments:

In Experiment 1, a bootstrapping approach was performed to obtain a surrogate condition and test whether entrainment was present or not. In Exp. 2 and 3, this seems initial condition is somewhat lifted: it is not consistently described whether significant entrainment or used as a factor in the analysis, for instance when the different entrainment frequencies are estimated (Fig. 3 b, c, d). Please include a statement about the general approach to your surrogate conditions in the methods.

**RE: we have included a statement about the general surrogate approach at the end of the methods section.**

*Pages 40-41, lines 1306-1319: “Surrogate distributions.*

*To assess the significance of behavioral entrainment to the FM stimulus in Experiment 1, surrogate datasets were generated by shuffling detection labels (1/0) across trials while keeping the FM stimulus phase fixed. To evaluate the effect of tACS on behavioral entrainment to sounds, surrogate distributions were created by shuffling the tACS phase lag relative to the sound.*

*In Experiment 2, the effect of each tACS frequency was assessed by comparing it to a baseline condition that estimated the strength of rhythmic perception during sham for each frequency. Additionally, to estimate the effect of tACS in the absence of a baseline oscillator, surrogate distributions were generated by shuffling detection labels (1/0) across trials while keeping the tACS phase fixed.*

*No surrogate distributions were used in Experiment 3, as the goal was to determine whether neural and behavioral entrainment were affected by the FM frequency, which was analyzed using a linear model. More details on these analyses are provided within each experiment.”*

2. Experiment 1:

ll 61:

"This is important because tACS effects are often weak, and
without quantifying the relative contributions of auditory and electrical rhythms to entrainment, we cannot know how strongly tACS can push brain rhythms around while it is competing with rhythmic sensory stimulation."

This statement seems to assume independent effects, but how about interactions, e.g. ceiling effects?

**RE: We agree with the reviewer that significant interactions may occur during concurrent tACS and auditory stimulation. Indeed, ceiling effects and nonlinear interactions have been previously described and are expected. This is precisely why we tested both modalities independently and in combination—to provide a clearer picture of how tACS influences entrainment to auditory stimuli and vice versa. We have added a sentence to the introduction explicitly stating that tACS and auditory rhythms can interact.**

*Page 3, lines 64-66: “We do not assume entrainment to electrical and auditory rhythms to be independent; significant interactions, such as ceiling effects and other nonlinear dynamics, are likely to occur (Krause et al., 2022).”*

3. l. 128: "No significant difference in the gap size thresholds was observed between the
   modulation depths."

That is indeed surprising. Please provide Bayes Factors to allow the reader to assess whether we are looking at a solid null-effect versus an inconclusive effect.

**RE: We have computed the Bayes factor which equals 0.389 suggesting that the evidence favors the null effect. This information has been added to the manuscript.**

*Page 6, line 131: “Bayes factor = 0.389”*

*Page 41, lines 1322-1323: “Bayes factor analyses were conducted using the* ***BayesFactor*** *package for MATLAB. (Krekelberg, 2025)”*

4. Fig. 1 is a bit crowded, even though the panels are clear and easy to understand with the caption. Maybe move panels i - l to a new figure?

**RE: Following the reviewer’s suggestion, we have moved panels i-l to a new figure (Figure 2). Consequently, all subsequent figure numbers have been shifted by one.**

5. H: the realignment procedure should be described for better clarity. I do not see a semi-transparent line in panel h, and I do not understand why these values were excluded.

**RE: The semitransparent line was in previous panel i) “EntAmp-FM as a function of tACS separated by modulation depth. The tACS lags correspond to the individually realigned lags; phase zero represents the individual optimal tACS lag estimated in (h). EntAmp-FM values from the realigned peaks and troughs (semi-transparent) were excluded from further analyses.” There might have been a confusion because we cite panel h when describing panel i. This should be clearer now as the two panels are in different figures, as suggested by the reviewer. The plot of reference is now Fig. 2b and the legend states: “b) EntAmp-FM as a function of tACS separated by modulation depth. The tACS lags correspond to the individually realigned lags; phase zero represents the individual optimal tACS lag estimated in (Fig. 1h). EntAmp-FM values from the realigned peaks and troughs (semi-transparent) were excluded from further analyses.”**

**For the realignment procedure, first we estimated the optimal tACS phase lag. We then found the tACS lag bin into which the individual optimal tACS phase fell, set this bin as zero phase and wrapped the other phases around. Since hit rates at the realigned optimal phase are the highest and at the realigned trough the lowest, comparing these values against each other would be trivial. Assuming that tACS did modulate *entAmp-FM* sinusoidally, we expected the two bins adjacent to the peaks and trough be significantly different from each other. This and similar realignment procedures have been previously used by our group and others ((Cabral-Calderin et al., 2024; Riecke et al., 2015). To illustrate the realignment better, we now provide a single participant example in figure 2a.**

*Page 8, Fig 2a:*

***Fig. 2. Experiment 1, tACS effects.*** *a) Realignment procedure. The optimal tACS lag (orange asterisk) was determined from the cosine function fitted to the EntAmp-FM by tACS phase-lag data (dashed gray line). The phase bin closest to the optimal phase was identified (pink asterisk). This bin was set to zero phase and the remaining phases were wrapped around. The new realigned data is shown in blue.*

6. Same for the description in l. 177, why would maintaining the values at the realigned peaks lead to a bias? If I understand correctly, only the phase lags were aligned, but this should not change the amplitude, correct?

**RE: The phase realignment does affect the amplitude parameter since the two are inherently linked. In the figure above, the *entAmp-FM* value at the bin closest to the optimal tACS phase is approximately 0.31. Previously, this corresponded to a phase lag of −2π/3. Now, with phase realigned to zero, *entAmp-FM* at zero phase becomes 0.31 and *entAmp-FM* at −2π/3 is about 0.24.**

**We have clarified this in our previous response and provided a new plot as an example. We hope this makes the procedure clearer.**

7. Experiment 2:

The analyses described in lines 265 and follows, p. 11, test for entrainment by tACS with respect to an imaginary oscillator that is already active.

How about the assumption that an internal oscillator is silent (in line with the Arnold-tongue approach), prior to tACS stimulation. This would mean using a different baseline, for instance phase-scrambled surrogates as in Experiment 1. Please explain why this approach was not used.

**RE: We tested entrainment relative to an active imaginary oscillator that is already active due to the existent literature that showing rhythmic fluctuations in perception that suggest that attention samples stimuli rhythmically (Fiebelkorn & Kastner, 2019; Landau & Fries, 2012). We considered this approach to be more rigorous that assuming no oscillation at all. Motivated by the reviewer’s comment we have incorporated also the possibility that there is no active oscillator at the stimulation frequency. As in experiment 1, we generated 1000 surrogate datasets per participant and frequency by randomizing the single-gap accuracy values (0,1) while preserving their tACS phase bin labels. The same cosine fits were done as for the original data and the real *entAmp-tACS* values were z-scored using the individual surrogate distributions. One-sample t-tests were performed to evaluate the significance of the z-scored *entAmp-tACS* values. No significant effect was observed at the group level for any of the frequencies (all t(23) < 0.5, all p>0.62, uncorrected). We have added this to the results and methods section**

*Page 12, lines 353-359: “To estimate the effects of tACS phase on gap detection relative to chance, surrogate distributions were created as in Experiment 1, by shuffling the gap accuracy values (1/0) across trials while keeping the tACS phase lag fixed. The same cosine fits were done as for the original data and the real entAmp-tACS values were z-scored using the individual surrogate distributions. One-sample t-tests were performed to evaluate the significance of the z-scored entAmp-tACS relative to chance. No significant effect was observed at the group level for any of the frequencies (all t_(23)_ < 0.5, all p>0.62, uncorrected).”*

*Pages 36-37, lines 1191-1196: “As in Experiment 1, surrogate distributions were created for each participant and frequency by shuffling the gap accuracy values (1/0) across trials while keeping the tACS phase lag fixed. The entAmp-tACS values were z-scored using individual surrogate distributions, and one-sample t-tests were performed to evaluate the significance of the z-scored entAmp-tACS values.”*

8. Fig. 2c: why are the amplitudes so different for the cosine fits across different tACS frequencies?

**RE: The cosine fits were done independently by participant and tACS frequency. The amplitude parameter is the result from the fit itself. As such, the amplitude differences across tACS frequencies suggest that at the individual level, behavioral entrainment to tACS was different across conditions.**

9. Ll 277: what is with the participants that fall outside the lower limit of the CI, aren't they noteworthy, too?

**RE: We have changed this plot and now show tACS effects relative to baseline, i.e., (entAmp-tACS-baseline)/baseline. We believe this plot to be more intuitive to the reader. Some participants show a positive while others show a negative tACS effect. Our interpretation is that in the latter case, tACS had a negative effect, likely due to a mismatch with the resonance frequency or ceiling effects—i.e., rhythmic behavioral fluctuations were already high at baseline, and tACS disrupted them. This section in the results now reads as follows:**

*Page 13, lines 384-395: “Thus, at the group level, we did not observe stronger behavioral modulation during tACS compared to baseline. To better appreciate the effect of tACS at the individual level, entAmp-tACS values were normalized to the baseline as (entAmp-tACS-baseline)/baseline (Fig. 3e). Inspection of the data shows high inter-individual variability with some participants showing positive and other a negative tACS effect. Closer inspection of the data showed that 17/24 participants showed a positive tACS effect that was > 1, for at least one of the tACS frequencies, meaning that entAmp-tACS was at least twice the baseline amplitude of rhythmic fluctuations at that frequency. The latter suggests that gap performance was modulated by tACS for part of the sample, but the relevant frequency was different across participants (see next section). On the opposite site, the negative tACS effects could potentially be due to a mismatch with the resonance frequency or ceiling effects—i.e., rhythmic behavioral fluctuations were already high at baseline, and tACS disrupted them.”*

10. In line with the upper point, I don't quite understand the surrogate condition here.

**The initial Fig. 2d showed the surrogate conditions as a continuous signal across frequencies representing the noise floor of behavioral entrainment, i.e., the expected amplitude of periodic fluctuations of gap detection performance independent of tACS. This was estimated by fitting cosine functions to the sham data from each participant, assuming an oscillator resetting at the stimulus onset at a given frequency. This analysis was conducted not only for the frequencies at which tACS was applied but also for all frequencies between 0.1 and 8 Hz, in 0.1 Hz steps, to provide a more comprehensive estimation of the probability of rhythmic fluctuations in behavior across the full delta/theta range. For each frequency, the mean amplitude of imaginary entrainment in the sham condition at that specific frequency, along with the standard error of the mean were shown.**

**Based on the reviewers’ comments, we realized that the figure and analysis description were not clear enough. We have clarified the analysis more and instead of “imaginary entrainment” we now call it “baseline”. We have also removed the plot from the figure and now show instead the change in behavioral entrainment during tACS relative to the baseline, i.e., (entAmp-tACS -baseline)/ baseline, which we hope is more intuitive. We also added a new panel illustrating the procedure (Fig. 3c).**

*Pages 11-12, lines 328-335: “c) For each tACS frequency, gaps were grouped based on tACS phase. Cosine functions were fitted to the data to estimate the amplitude of behavioral entrainment to tACS. Gap detection performance at sham was used to establish a baseline level of rhythmic fluctuations. We assumed an oscillator that resets at each stimulus onset during sham. Gaps were grouped according to these hypothetical oscillators, and cosine functions were fitted to the resulting data. This was done for the four frequencies used for tACS stimulation. The amplitude parameter from these fits served as the baseline for analyzing tACS effects. In the figure, this is illustrated for the 2 Hz tACS condition.”*

11. Exp. 2, Oscillator model, Fig. 3b: I first tried to read from the plot in 3B the tACS frequencies at which the highest entrainment amplitude was found, which seems to match the resulting resonance frequency in some, but not all cases, e.g. the left-most plot in the top row. Then I understood that the selection of the best model (individual resonance frequency) was done based on correlation of the entAmp-tACS and the model-produced values, across tACS frequencies, hence for 4 values? It would help to show these correlations / their distributions, instead of plotting entrainment amplitude on the y-axis.

**RE: Following the reviewer’s suggestion, we have removed this plot and now provide the individual plots showing the correlations between the model and the empirical data as a function of the ongoing oscillator frequency (Figure 4c).**

*Pages 14-15, lines442-458: “c) Individual data showing the correlation between the model output — change in entrainment relative to baseline — and the amplitude of behavioral entrainment to tACS – (entAmp-tACS-baseline)/baseline- as a function of the model’s ongoing oscillator resonance frequency. The boxes at the top of each plot display the effect of tACS in Experiment 2 for each individual participant. Colored squares indicate positive (top) and negative (bottom) tACS effects relative to baseline. Red squares highlight participants with a tACS effect greater than 1, meaning entAmp-tACS is at least twice the baseline amplitude. RF: resonance frequency, red asterisks.”*

12. Also, the fit between the model and the data is quite striking - how do you explain that the model fits the actual amplitudes so well, for instance in participants like the fifth from the left in the bottom row, whose amplitude curve has a non-linear shape?

**RE: Initially, the fit plots displayed z-score values. While the model did not fit the actual amplitudes, it followed the overall pattern. These plots have now been removed in accordance with the reviewer’s suggestion, and we instead provide correlation plots. Additionally, the analysis in this figure has been revised. Previously, correlations between the model and the data were based on amplitude parameters without baseline correction. The current approach correlates values after baseline correction, providing a more accurate representation of tACS entrainment. See the figure legend above for further details.**

*Page 15, lines 471-474: “The amplitude of entrainment to tACS is then quantified as (tACS amplitude−baseline amplitude)/baseline amplitude. This change in oscillation amplitude relative to baseline is subsequently correlated with the change in behavioral modulation due to tACS, calculated as (entAmptACS−baseline)/baseline.”*

13. The analyses of individual oscillator frequencies are conducted on all participants, even though only 16 / 24 showed significant amplitude modulation by tACS (Fig. 2d). Did the results differ for those participants, and what was the reasoning to include them in the estimation of the resonance frequency?

**RE: The reasoning for including all participants was to determine whether response patterns could be predicted by the mismatch between individual resonance frequency and tACS. We have now added information about each participant’s tACS effect alongside the model-behavior correlation in Figure 4c (see answer to next comment). This allows the reader to better appreciate how the estimated resonance frequency relates to whether a participant responded positively or negatively to tACS at a specific frequency.**

*Page 15, lines 477-480: “Note that we included all participants here regardless of whether they responded positively or negatively to tACS to determine whether response patterns could be predicted by the mismatch between individual resonance frequency and tACS.”*

14. As presented here, the estimation of the resonance frequency does not seem to have a baseline condition, which allows to assess whether there is resonance at all. In other words, a resonance frequency is always found, even if there might be no entrainment to the tACS, right? Is there a way to test if there is resonance in the first place?

**RE: The estimation of the resonance frequency now includes a baseline condition. The model starts with an ongoing oscillator at a specific frequency (0.1–8 Hz, in 0.1 Hz increments) and simulates how this oscillator is influenced by tACS at each frequency of interest (0.8, 2, 3.2, and 4.4 Hz), depending on the relative strength of tACS to the ongoing oscillation. The amplitude of entrainment to tACS is then quantified as (tACS amplitude−baseline amplitude)/baseline amplitude. This change in oscillation amplitude relative to baseline is subsequently correlated with the change in behavioral modulation due to tACS, calculated as (entAmptACS−baseline)/baseline. The resonance frequency is defined as the ongoing oscillator frequency at which the model's tACS-driven entrainment change shows the highest correlation with tACS-driven behavioral modulation in the experimental data. As the reviewer correctly pointed out, a resonance frequency will always be found, as the model assumes the presence of an ongoing oscillator with a preferred frequency that can be entrained by tACS. However, it is important to note that the correlation values are highly significant (>0.89).**

**We have clarified this information in the results section. To make this point clearer, we now include an example of the model results for a 2 Hz ongoing oscillator in Figure 4a and provide the correlation values between the model and experimental data for each participant in Figure 4c. Additionally, Figure 4c now indicates whether each participant exhibited a positive or negative tACS effect in the experimental data.**

*Page 15, lines 466-477: “The model simulates an ongoing oscillator at a specific resonance frequency (0.1 and 8Hz in steps of 0.1 Hz) and models how this oscillator is influenced by tACS at each frequency of interest (0.8, 2, 3.2, and 4.4 Hz), depending on their frequency mismatch and the relative strength of tACS to the ongoing oscillation (Fig. 4a-b). TACS amplitude took on values from 0 to 100% of the baseline oscillation’s amplitude, in steps of 10%, which corresponds to physiologically relevant conditions (Krause et al., 2022). The amplitude of entrainment to tACS is then quantified as (tACS amplitude−baseline amplitude)/baseline amplitude. This change in oscillation amplitude relative to baseline is subsequently correlated with the change in behavioral modulation due to tACS, calculated as (entAmptACS−baseline)/baseline. The resonance frequency is defined as the ongoing oscillator frequency at which the model's tACS-driven entrainment change shows the highest correlation with tACS-driven behavioral modulation in the experimental data. (Fig. 4c).”*

***Fig. 4. Experiment 2: Oscillator model.*** *a)* *Model results for a 2 Hz ongoing oscillator at each tACS frequency, shown for three different k values -tACS strength relative to the ongoing oscillator amplitude. For illustration purposes, the tACS signal in the plots is phase-shifted by 180 degrees relative to the ongoing oscillation to better visualize entrainment. The percentage at the top of each plot represents the change in entrainment due to tACS -(entrainment amplitude during tACS-baseline)/baseline. b) Output of the oscillator model showing the change in entrainment by tACS -(entrainment amplitude during tACS-baseline)/baseline- as a function of the tACS amplitude relative to the baseline, and the frequency mismatch between the tACS frequency and the resonance frequency of the internal oscillator. c) Individual data showing the correlation between the model output—change in entrainment relative to baseline—and the amplitude of behavioral entrainment to tACS – (entAmp-tACS-baseline)/baseline- as a function of the model’s ongoing oscillator resonance frequency. The boxes at the top of each plot display the effect of tACS in Experiment 2 for each individual participant. Colored squares indicate positive (top) and negative (bottom) tACS effects relative to baseline. Red squares highlight participants with a tACS effect greater than 1, meaning entAmp-tACS is at least twice the baseline amplitude. RF: resonance frequency d) Predicted individual resonance frequencies. Each dot represents a single participant. e) Dendrogram showing the clustering results. Each color represent a different cluster. f) EntAmp-tACS values as a function of tACS frequency grouping of participants based on their cluster identities in (e). Spread plots at the top show the predicted individual resonance frequencies for each participant in the cluster corresponding to the plots at the bottom. Each dot represents a single participant. Box plots show median (horizontal black lines), mean (black cross), 25th and 75th percentiles (box edges) and extreme datapoints not considered outliers (+/–2.7σ and 99.3 percentiles, whiskers).*

15. Figure 3a: what is the unit of the change of entrainment, and how to interpret the y-axis?

**RE: The change in entrainment is expressed as a percentage relative to baseline. The model uses arbitrary units rather than specific measurement units. The baseline oscillation had an amplitude of ~0.45 a.u. across frequencies, and the change in entrainment is calculated as (amplitude during tACS−baseline)/baseline. We have clarified this information in the figure (now Figure 4b) and in the Methods section**.

*Page 37, lines 1221-1222: “The amplitude of the ongoing oscillation was ~0.45 across frequencies.***”**

*b) Output of the oscillator model showing the change in entrainment by tACS -(entrainment amplitude during tACS-baseline)/baseline- as a function of the tACS amplitude relative to the baseline, and the frequency mismatch between the tACS frequency and the resonance frequency of the internal oscillator*

16. Experiment 3:

If I understood correctly, the duration of the stimulation was constant across the different frequencies, i.e. there are more cycles in the faster condition, which might bias the measure of resultant vector length and explain the linear increase in Fig. 6c.

**RE: The reviewer is right that there are more cycles in the faster condition. However, we don’t believe this to bias the resultant vector length (VL) estimation. As stated in the Methods section (page 35, lines 964-966) the resultant vector length was computed on the phase angles from the complex FFT output across trials, separately for each frequency and electrode. That is, while there are more cycles in the faster condition, by using only one phase value per trial the same amount of data in going into the VL calculation. Moreover, the VL shown in previous Fig. 6c is the normalized value, i.e., the VL observed at each frequency during the FM stimulus presentation at the same frequency was divided by the mean VL recorded at the same frequency when the FM stimulus was presented at all other frequencies. Both VL values are computed for the same EEG frequency, by dividing one by the other we control for any bias on estimating this parameter. See Page 38, lines 1046-1053. Note however that based on other comments from the reviewer this figure has been removed.**

17. l. 550, p. 21, Discussion:

The authors argue that the smaller sample size compared to their previous study might have contributed to the absence of a significant effect of entrainment in this work. It would be helpful for future studies to provide the relevant numbers (participants, trials) in the discussion paragraph. Would it be possible to compute the measures from the previous study on similar trial numbers?

**RE: We sincerely thank the reviewer for this suggestion. We agree that providing this information is valuable, as it can guide future studies in planning tACS experiments with similar designs. To address this, we repeated our analysis on the previous dataset, using a sample size comparable to the present study. While the overall pattern of results remained the same, significance was not reached when compared to the surrogate distribution. Specifically, we ran this analysis 500 times, randomly sampling N=27 from the 42 participants in our previous study. For completeness, we extended the analysis across sample sizes N=27 to N=40 and quantified the probability of obtaining a significant effect for each condition as a function of N. Importantly, not only the number of participants but also the number of sessions influences the probability of detecting a significant effect. The significance threshold is reached more quickly when using data from both sessions compared to using data from a single session. We have added this information to the discussion section and included a new supplemental figure (Figure S3).**

*Page 24, lines 807-814: “To clarify this point, we conducted the statistical analysis using the pre-processed data from our previous study (Cabral-Calderin et al., 2024), randomly selecting different sample sizes and using either one or both sessions. Figure S4 confirms that the probability of observing a significant effect increases with sample size and is higher when data from multiple sessions is included. This analysis suggests that a minimum of 32 participants and two sessions are required to detect a significant difference in behavioral entrainment to the FM stimulus between positive and negative half-cycles of tACS with 0.95 probability. However, this number may vary depending on the specific task.”*

***Fig. S4. Effect of sample size on estimating tACS effects.*** *a) Histograms showing the distribution of p-values obtained from estimating tACS effects in 500 random samples of N = 27 using the data from (Cabral-Calderin et al., 2024). The distributions are shown for sample of N = 27 using only one session data or the average across two sessions. b) Probability of observing a significant tACS effect of each contrast of interest as a function of the sample size (N =27-42). Note that for N = 42, only one independent sample can be drawn and only 42 different samples could be drawn for N = 41. Similar to (a), the analysis was performed using data from only one session or using the average across two sessions.*

18. The speculative point made about the similarity of the trajectories in Figure 6 an and b is not very apparent from the Figure. I would consider removing this point, to make the paper a bit less complex.

**RE: We thank the reviewer for their comment. We agree that this point may not have been apparent from the original figure. Based on the reviewer’s feedback and a comment from Reviewer 2, we have revised the data visualization and now display the amplitude of entrainment across tasks for each participant. We still consider this an important question and prefer to keep it in the manuscript. However, we have rephrased and condensed the section, and the updated figure has been moved to the supplementals (Figure S4). The revised section now reads:**

*Page 20, lines 629-641: “Optimal frequency for behavioral and neural entrainment do not overlap*

*We showed that the optimal frequency for entrainment to both tACS and FM stimuli varies across participants. To close the loop, we asked if the preferred frequencies were stable within an individual, regardless of the modality of the rhythmic stimulus. Figure S3 shows the amplitude of behavioral and neural entrainment to tACS and the FM stimulus, respectively, for the 12 participants who took part in both Experiments 2 and 3. While some participants exhibit similar response profiles across tasks and measures, most show different frequency preferences (i.e., the frequency with the highest entrainment) across tasks. This suggests that the optimal entrainment frequency may depend on the stimulation modality. However, note that we tested only four frequencies and included only 12 participants in this analysis. Unfortunately, it was not possible to re-recruit more participants for testing because of the unavailability of participants and relocation of the research group. This remains speculative and requires further investigation with larger sample sizes and measuring more stimulation frequencies.”*

***Fig. S3. Linking individual differences in response profiles across modalities.*** *The plots display entrainment amplitude as a function of frequency for Experiments 2 and 3. In Experiment 2, behavioral entrainment to tACS is quantified as (entAmp-tACS−baseline)/baseline, while in Experiment 3, behavioral and neural entrainment to FM sounds are represented by entAmp-FM and normalized vector length (VL), respectively. Data is shown for the twelve participants who took part in both experiments. Black dashed squares highlight participants with a tACS effect greater than 1 for at least one frequency. Red dashed squares indicate participants with a positive tACS effect for at least one frequency, but less than* 1.

*Pages 29-30, lines 948-951: “When examining individual response profiles across tasks, we observed that while a few participants exhibited similar response patterns across tasks and measures, most displayed different frequency preferences (i.e., the frequency with the highest entrainment) across tasks.”*

**References**

Baltus, A., Wagner, S., Wolters, C. H., & Herrmann, C. S. (2018). Optimized auditory transcranial alternating current stimulation improves individual auditory temporal resolution. *Brain Stimulation*, *11*(1), 118-124. <https://doi.org/https://doi.org/10.1016/j.brs.2017.10.008>

Brodbeck, C., Jiao, A., Hong, L. E., & Simon, J. Z. (2020). Neural speech restoration at the cocktail party: Auditory cortex recovers masked speech of both attended and ignored speakers. *PLoS Biol*, *18*(10), e3000883. <https://doi.org/10.1371/journal.pbio.3000883>

Cabral-Calderin, Y., Anne Weinrich, C., Schmidt-Samoa, C., Poland, E., Dechent, P., Bahr, M., & Wilke, M. (2016). Transcranial alternating current stimulation affects the BOLD signal in a frequency and task-dependent manner. *Hum Brain Mapp*, *37*(1), 94-121. <https://doi.org/10.1002/hbm.23016>

Cabral-Calderin, Y., & Henry, M. J. (2022). Reliability of Neural Entrainment in the Human Auditory System. *J Neurosci*, *42*(5), 894-908. <https://doi.org/10.1523/JNEUROSCI.0514-21.2021>

Cabral-Calderin, Y., van Hinsberg, D., Thielscher, A., & Henry, M. J. (2024). Behavioral entrainment to rhythmic auditory stimulation can be modulated by tACS depending on the electrical stimulation field properties. *Elife*, *12*. <https://doi.org/10.7554/eLife.87820>

Cabral-Calderin, Y., Williams, K. A., Opitz, A., Dechent, P., & Wilke, M. (2016). Transcranial alternating current stimulation modulates spontaneous low frequency fluctuations as measured with fMRI. *Neuroimage*, *141*, 88-107. <https://doi.org/10.1016/j.neuroimage.2016.07.005>

Doelling, K. B., Arnal, L. H., Ghitza, O., & Poeppel, D. (2014). Acoustic landmarks drive delta-theta oscillations to enable speech comprehension by facilitating perceptual parsing. *Neuroimage*, *85 Pt 2*(0 2), 761-768. <https://doi.org/10.1016/j.neuroimage.2013.06.035>

Doelling, K. B., & Poeppel, D. (2015). Cortical entrainment to music and its modulation by expertise. *Proc Natl Acad Sci U S A*, *112*(45), E6233-6242. <https://doi.org/10.1073/pnas.1508431112>

Eddins, D. A., Hall, J. W., 3rd, & Grose, J. H. (1992). The detection of temporal gaps as a function of frequency region and absolute noise bandwidth. *J Acoust Soc Am*, *91*(2), 1069-1077. <https://doi.org/10.1121/1.402633>

Fiebelkorn, I. C., & Kastner, S. (2019). A Rhythmic Theory of Attention. *Trends Cogn Sci*, *23*(2), 87-101. <https://doi.org/10.1016/j.tics.2018.11.009>

Golumbic, E. M. Z., Ding, N., Bickel, S., Lakatos, P., Schevon, C. A., McKhann, G. M., Goodman, R. R., Emerson, R., Mehta, A. D., Simon, J. Z., Poeppel, D., & Schroeder, C. E. (2013). Mechanisms Underlying Selective Neuronal Tracking of Attended Speech at a "Cocktail Party". *Neuron*, *77*(5), 980-991. <https://doi.org/10.1016/j.neuron.2012.12.037>

Green, D. M., & Forrest, T. G. (1989). Temporal gaps in noise and sinusoids. *J Acoust Soc Am*, *86*(3), 961-970. <https://doi.org/10.1121/1.398731>

Grose, J. H., Buss, E., & Hall, J. W., 3rd. (2008). Gap detection in modulated noise: across-frequency facilitation and interference. *J Acoust Soc Am*, *123*(2), 998-1007. <https://doi.org/10.1121/1.2828058>

Henry, M. J., Herrmann, B., & Obleser, J. (2014). Entrained neural oscillations in multiple frequency bands comodulate behavior. *Proc Natl Acad Sci U S A*, *111*(41), 14935-14940. <https://doi.org/10.1073/pnas.1408741111>

Henry, M. J., & Obleser, J. (2012). Frequency modulation entrains slow neural oscillations and optimizes human listening behavior. *Proc Natl Acad Sci U S A*, *109*(49), 20095-20100. <https://doi.org/10.1073/pnas.1213390109>

Horton, C., Srinivasan, R., & D'Zmura, M. (2014). Envelope responses in single-trial EEG indicate attended speaker in a 'cocktail party'. *J Neural Eng*, *11*(4), 046015. <https://doi.org/10.1088/1741-2560/11/4/046015>

Krause, M. R., Vieira, P. G., Thivierge, J. P., & Pack, C. C. (2022). Brain stimulation competes with ongoing oscillations for control of spike timing in the primate brain. *PLoS Biol*, *20*(5), e3001650. <https://doi.org/10.1371/journal.pbio.3001650>

Krekelberg, B. (2025). bayesFactor (<https://github.com/klabhub/bayesFactor>), GitHub. Abgerufen15. Januar 2025. In.

Landau, A. N., & Fries, P. (2012). Attention samples stimuli rhythmically. *Curr Biol*, *22*(11), 1000-1004. <https://doi.org/10.1016/j.cub.2012.03.054>

Mizuno, C., Schwartz, J. L., & Cazals, Y. (1994). Periodicity of long-term context can influence gap detection. *Hear Res*, *78*(1), 41-48. <https://doi.org/10.1016/0378-5955(94)90042-6>

Nozaradan, S., Peretz, I., & Keller, P. E. (2016). Individual Differences in Rhythmic Cortical Entrainment Correlate with Predictive Behavior in Sensorimotor Synchronization. *Sci Rep*, *6*, 20612. <https://doi.org/10.1038/srep20612>

Numssen, O., Kuhnke, P., Weise, K., & Hartwigsen, G. (2024). Electric-field-based dosing for TMS. *Imaging Neuroscience*, *2*, 1-12. <https://doi.org/10.1162/imag_a_00106>

Peelle, J. E., & Davis, M. H. (2012). Neural Oscillations Carry Speech Rhythm through to Comprehension. *Front Psychol*, *3*, 320. <https://doi.org/10.3389/fpsyg.2012.00320>

Riecke, L., Sack, A. T., & Schroeder, C. E. (2015). Endogenous Delta/Theta Sound-Brain Phase Entrainment Accelerates the Buildup of Auditory Streaming. *Curr Biol*, *25*(24), 3196-3201. <https://doi.org/10.1016/j.cub.2015.10.045>

Shailer, M. J., & Moore, B. C. (1985). Detection of temporal gaps in bandlimited noise: effects of variations in bandwidth and signal-to-masker ratio. *J Acoust Soc Am*, *77*(2), 635-639. <https://doi.org/10.1121/1.391881>
